# The Hospital. Nursing Section

**Published:** 1904-07-09

**Authors:** 


### The Hospital.

**Nursing Section.**

Contributions for this Section of "The Hospital" should be addressed to the Editor, "The Hospital" Nursing Section, 28 & 29 Southampton Street, Strand, London, W.C.

No. 928.—Vol. XXXVI. *SATURDAY, JULY 9, 1904.*

### Notes on News from the Nursing World.

### RUSSIAN NURSES IN PERIL.

It will be recollected that during the campaign in South Africa many of our nurses carried on their duties under great drawbacks, several of them being almost flooded out of their tents. Their experience, however, was never so perilous as that of the Sisters of Mercy who are nursing Russian soldiers. The heavy rains in the Far East have had the most disastrous effects. A water wave, suddenly rolling down the valley at Ying-Kow, swept away the barracks of the Red Cross and the tents of the hospitals, and the Sisters of Mercy were with difficulty rescued by the soldiers, who had to take them up in their arms and stumble along as best they could. The dangers of the situation may be judged by the fact that all the tents were so filled with water that they had to be cut open with sabres, and that horses and other animals were swept away. About 14 men are said to have been drowned; but so far as it is known, the Sisters of Mercy have all escaped with their lives.

### THE CENTRAL MIDWIVES' BOARD AT LOGGERHEADS.

The proceedings at the first meeting of the Central Midwives' Board, to which representatives of the medical and nursing papers were admitted, were far from satisfactory from a nursing point of view. The principal question before the Board was the consideration of an examination scheme drafted by Dr. Cullingworth. A resolution was moved by Mr. Ward Cousins to the effect that the members of the medical profession only should be eligible for appointment as examiners, and Mr. Parker Young, who followed in support of the motion, declared that, in his opinion, nursing can only properly be taught by medical practitioners. Dr. Cullingworth took an entirely different view, and having urged that the appointment of examiners being entirely in the discretion of the Board they should be free to appoint whom they would, said that most of them knew women competent for the work. Subsequently, Mr. Parker Young made some remarks not complimentary to the lady members of the Board, and Dr. Sinclair considered it not beneath his dignity to indulge in some observations about "highly gifted chambermaids." We are glad to learn that the resolution, which was quietly but effectively opposed by Miss Wilson, was lost; but it is a matter for regret that some of the medical members of the Board should adopt an attitude which exposes them to the suspicion of being actuated by a petty jealousy of which they ought to be incapable.

### PARLIAMENT AND REGISTRATION.

It appears that the appointment of a Select Committee of the House of Commons to consider the expediency of providing for the registration of nurses has raised hopes that legislation may be anticipated at an early date. Apart from the fact that the Committee will not proceed far with this inquiry before the Prorogation, and the prospects of a speedy dissolution of Parliament, it is only in the event of their report being of a very conclusive and unanimous character that legislation is likely to be initiated in consequence. The reference to a Select Committee has shelved so many questions that we doubt whether the advocates of registration would not have been wiser if they had decided to take their chance with their Bills in the ballot next session.

### NURSES OF THE SAME PATTERN.

In his speech at a drawing-room meeting under the auspices of the Nurses' National Total Abstinence League, reported in another column, Sir William Collins urged his hearers to use some of their scanty leisure to study some social reform. He expressed his belief that a central institute or a central training college, giving nurses an opportunity to specialise, would be excellent, "as there seemed to be too many nurses of the same pattern." We think that there is no doubt about the accuracy of Sir William's conclusion. It is one of the drawbacks of life in a hospital that the institution is too often regarded as the hub of the universe; and matrons who encourage their nurses to enlarge their ideas by taking an interest in outside matters, and by not talking about their work when they are off duty, do something towards counteracting this evil. As Sir William Collins suggests, a greater variety of aspiration is frequently needed—"something beyond the desire to nurse a great surgical case." His proposal for the establishment of a central institute or training college may some day become a practical question; and meanwhile nurses who desire to keep out of the groove which tends to render them of one pattern cannot do better than avail themselves of any opportunities that are offered them to increase their knowledge of literature, science, art, music, or of foreign countries, by means of travelling which, while not diverting their attention from their work, cannot fail to broaden their views and elevate their aspirations.

### NURSING ASSOCIATIONS AND THE POWER OF SUING.

An interesting issue arose in Southmolton County Court the other day. The honorary secretary of the Southmolton Nursing Association sued a farmer in the neighbourhood for £17 16s. 6d., which it was alleged was due to the association for the nursing and cost of maintenance of a patient in the institution who was injured by an accident on the defendant's farm. At the outset the counsel for the defendant raised the objection that the society, being supported by voluntary contributions and neither incorporated nor registered, had no power to sue. He also urged that the defendant could not be sued because it could not be proved that he had received any benefit. Both these points were overruled by Judge Beresford, who, however, after giving judgment in favour of the nursing association, granted permission to appeal. The judgment, apart from the technical issue, appears to us to have been the inevitable result of the very clear evidence of the doctor who attended the patient and the nurse in charge of the hospital. But we think that leave to appeal was wisely permitted. It is of great importance to nursing organisations similar to that of Southmolton throughout the country that it should be authoritatively decided whether they have, or have not, the power to sue.

### MARRIAGE OF A MATRON.

On Tuesday Miss Florence Strange, who has been matron of Chalmers Hospital, Edinburgh, for four years, was married at St. Barnabas, Kensington, to Mr. Yardley, of Streatham. Prior to her departure from Edinburgh Miss Strange was presented with a handsome silver jug by the directors, with numerous gifts by past and present nurses, and with a fish-slice by the domestic staff.

### DISTRICT NURSING IN AUSTRALIA.

At a recent meeting of the Royal Victorian Trained Nurses' Association in Melbourne a very interesting paper on district nursing was read by Sister Archer. The daily work of the district nurse at the Antipodes runs much on the same lines as that of the district nurse in England. The arrangements at the home in Carlton, which Miss Archer describes, are as follows :—There is accommodation for four nurses, the sister-in-charge, and a servant. Each nurse has a room to herself, or shares one large balcony room. There is a general sitting-room and dining-room, and a very small dispensary. Each nurse is paid £50 per annum, and, if she does not possess a bicycle, tram tickets are provided. The uniform is a blue print dress, cuffs, collar, straw hat with white band and red cross in front, and a cloak for outdoor use. Each nurse has her own district assigned to her, but those who have bicycles do not keep strictly to the boundaries. None but trained nurses are employed, and a small fee is usually, though not invariably, collected, the amount raised in this way being added to the general fund.

### THE NURSES OF HUDDERSFIELD INFIRMARY.

A NOTABLE ceremony took place last week at Huddersfield Infirmary. Several friends interested in the training of the nurses recently subscribed for a gold medal to be awarded annually to the nurse who comes out first in the examination for the year. The medal is a beautiful piece of workmanship, containing on the face an inlaid view of the Infirmary, the crest of the County Borough of Huddersfield, and the words "Awarded for Efficiency." On the reverse side the names of the winners are be inscribed. These names are also to be recorded upon an illuminated board placed in the hospital. There was keen competition amongst the nurses anxious to be the first holder of the medal, and it was gained by Nurse Wright. The presentation was accordingly made on Thursday afternoon by Mrs. Sandford, wife of the Vicar of Huddersfield, who in a graceful little speech congratulated Miss Wright and wished her much happiness and success in the most useful and honourable profession of nursing. Hearty applause greeted the winner of the medal as Mrs. Sandford pinned it upon her apron, testifying to the esteem in which she is held by her fellow-nurses. Mr. Edwards Watkinson, a member of the Infirmary Board, on behalf of the matron and the company present, thanked Mrs. Sandford for her presence and the gracious way in which she made the presentation. A garden party was subsequently given in the hospital grounds, bringing to an enjoyable termination the first of what should be a long series of presentations which will be looked forward to yearly with great eagerness by those nurses who receive their training at Huddersfield Infirmary.

### A NURSE AS GUARDIAN.

THE practical advantage of a nurse being a guardian of the poor was shown in the proceedings at a meeting of the Fylde Board last week. Miss Johnson, of Lytham, who for the last ten years has in conjunction with a St. Thomas's nurse carried on a hydropathic establishment at Lytham, is herself a nurse who received her training at one of the best provincial hospitals. Having been recently elected one of the Fylde Guardians, she lost no time in making it clear that she intends to be an active member. Her first action has been to bring before the Board an alleged scandalous state of affairs at the workhouse schools. Miss Johnson affirms that for some months children have been going to school with ringworm, that though suffering from ringworm they were walking about stone floors barefooted, and that the only excuse given for their attendance was that if they were kept at home for a day the school-attendance officer made a fuss. The house committee appear to have ignored Miss Johnson's allegations, but the Board took a different view of their importance and referred them back to the committee. The serious nature of the charges is obvious, and if they can be sustained, it will be necessary both in the interests of health and of economy for the guardians to prevent the possibility of a recurrence.

### THE SCHOOL NURSE IN PHILADELPHIA.

A REPORT of the result of the experimental appointment of a school nurse in the city of Philadelphia shows that in the five months she has been at work she paid 1,420 visits to the school-room, and 994 visits to the homes. She also sent 16 cases and took 14 to the dispensaries. The principal ailment in the school was pediculosis, but there were also cases of impetigo, ringworm, infected fingers, and eczema. The greatest difficulty encountered by the nurse was that of convincing the mothers of the necessity of giving each child a bath and clean clothes once a week. They had to be taught how to bath the children. But the testimony of the nurse is that the people endeavoured to the best of their ability to carry out all her suggestions in regard to hygiene, and altogether the effect of her work has been so marked that it is expected to influence other large cities in the United States who have no school nurse at present, to make a similar appointment.

### PRINCESS CHRISTIAN'S TRAINED NURSES.

The seventeenth annual report of Princess Christian's trained nurses, just issued by the committee, shows that the staff now consists of a lady superintendent, an assistant superintendent, five district Queen's nurses—including midwives—working in Windsor, and 25 private nurses; also four Queen's nurses in the branch districts of Eton, Chertsey, Addlestone, and Egham. During the year 392 cases were nursed by the private nurses and 28 refused; 31 were received at reduced fees, three gratis, and eight district patients were given massage free of charge. The private nurses also helped in the district work during the holidays of the district nurses. Five paying patients were nursed in the home. All the nurses had five weeks' holiday, and nine received a bonus on their earnings in addition to their salaries, which range from £30 to £40 with board and uniform. Of the nurses who left in the year one has been appointed matron of a hospital in the Isle of Wight, another has taken up a hospital appointment in South Africa, four have been engaged for district work in London, Darlington, and Clewer, one is married, and one was found unsuitable for the work.

### THE DIETARY OF POOR-LAW NURSES.

The Guardians of South Stoneham have received a communication from the Local Government Board with reference to a proposal that the nurses' dietary should be varied by the use of fish and poultry in place of meat. We are glad to learn that the Board have no objection to the variation, and that it is therefore now open to other guardians to vary the dietary in a manner which cannot fail to be beneficial to the nurses. Meat is far too often the only food given them for dinner, even in cases where the scarcity of fish or poultry does not provide a reason for not supplying them, at any rate, occasionally.

### AN IRON CONTRIBUTION BOX FOR ROMSEY.

At Romsey, in Hampshire, there was a unique little ceremony the other day. An iron contribution box, on which is painted the Geneva Cross, has been built into the wall of the nursing home garden, and it was formally uncovered by Mrs. Chichester. Before the Vicar of Romsey called upon her to perform the ceremony, he explained that the box had been placed in the wall to receive subscriptions from any one at the moment who might pass, and was reminded by the sight of the home of the excellent work it was doing in the town. Mrs. Chichester then pulled a white cord which hoisted a flag with a red cross in the centre, and which had previously hidden the box from the view of the public. Subsequently, Mr. Silence, the originator of the idea, described how the work had been done, and expressed his gratitude to the various persons who had gratuitously assisted him to carry it out. Another speaker, having pointed out that the box "was not one of those puny things which were pushed down every one's throat," said that, "the matron was the person who would empty the box, and the company present were the people who would fill it." They certainly made a respectable start, for the contributions in the box for the first day amounted to £3 8s. 4d. The experiment at Romsey may be commended to the managers of other nursing organisations. An iron box in a prominent position affords an excellent opportunity for persons who can only afford to give a little, or who desire to conceal their identity, to assist a deserving cause.

### QUEEN'S NURSES AT ACCRINGTON.

At the sixth annual meeting of the Accrington District Nursing Association the report was adopted. The number of visits paid by the three nurses during the year was 9,312, against 9,142 in the previous year, and the balance-sheet shows that the receipts exceeded the expenditure to the extent of £83. But this does not mean a gain, as the balance in the treasurer's hands when the year commenced was £132. The association would be in a stronger position with more collectors, each striving, as those already engaged in the work strive, to collect at least five pounds annually. The committee urge the importance of making the regular income adequate, so as to do away with the need for special appeals of all kinds. This is the right principle, and it seems to have already met with very encouraging support at Accrington.

### AN INADEQUATE GRANT.

The Caersws Board of Guardians have decided to contribute the sum of four guineas to the support of the Queen's nurses who work under the auspices of the District Nursing Association. They have been a long time making up their minds to even this small concession. For 12 years there has been a saving of rates in Newtown owing to the visits of the nurse to pauper patients in their own homes, and for seven years the nurse attached to the Llanidloes branch has been promoting a similar result on a smaller scale. Miss Lewis, the guardian who claimed a grant on the ground of justice, stated that last year the nurses by their visits saved the house from supporting 10 more paupers; and, while a little help, which admits the soundness of the principle, is better than none at all, it is a matter for regret that the Board considered it sufficient to vote two guineas for each branch.

### WELCOME OF A NURSE TO BATLEY.

A remarkably enthusiastic welcome has been accorded to Miss Ross, who has undertaken the work of a district nurse at Batley in connection with the branch of St. John's Ambulance Association. In honour of her arrival a picnic was held in the grounds at Woodhall, and a large company assembled on the occasion, including the Mayor and his wife. The Mayor, in the course of his speech, observed that in towns not half the size of Batley four or five nurses were fully employed, and we conclude from his comments and those of others at the gathering, that the appointment of Miss Ross, which has been so well received, is the prelude to the engagement of an adequate staff.

### GARDEN FÊTE AT SOUTHPORT.

The Southport and Birkdale District Nursing Society has derived substantial benefit from a garden fête and sale of work which has been held in the rectory grounds at Southport. Canon Denton Thompson, in opening the sale, spoke of the good results achieved by the Society, and pointed out that the committee urgently require fresh subscribers. This need, of course, still continued, but the amount realised by the garden fête was about £120.

### The Nursing Outlook.

"From magnanimity, all fear above;
From nobler recompense, above applause,
Which owes to man's short outlook all its charm."

### HOLIDAY TIME.

WITH the coming of July all our thoughts turn towards the country and the sea, and the majority of nurses begin to plan their holidays. The hospital nurse longs for some place where she can sleep and rest. She dreams of a bank of heather, or of a sandy shore where she can laze in the sun and the wind. The district nurse is generally more active in her desires. She plans foreign tours; she thinks chiefly of change. The private nurse wants to go home—to some circle of friends with whom she has no professional relation, and where she can forget cap and apron and the vagaries of the sick.

But it is not all of us who have a home to go to, and it is not always wisest to idle all our holiday away. It is generally acknowledged that the main temptation of a nurse's life is narrow-mindedness; to secure a large and healthy view of life in general, it is necessary to travel, to secure change of thought and scenery and food, to mix with those whose aims and works are different from our own.

And this can be obtained, and all bother of making arrangements ahead avoided, by taking advantage of one of the many public parties, or by joining a party of friends, who arrange for co-operative travel. There was a great objection to travelling in a party at one time, because a guide was considered a necessary adjunct, and the travellers were regarded as herds of ignorant sheep. But all this has been remedied, and nowadays parties break up into little groups, and are quite free to spend their time to their liking.

Take, for instance, one of the advertised five- or eight-guinea tours to Switzerland. You write beforehand and get a list, and mark the one you want; the arrangements for the journey out and for the first week or ten days are all made for you; you have nothing to do but to turn up at Charing Cross at the right hour. At the station you find a gentleman wearing a distinctive badge ready to advise you about your luggage, and to show you the train; you will probably find a sprinkling of clergy, medical men, teachers and their wives, going with the party, and the usual percentage of lively old maids, who are beginning to live at 50! You travel straight through, the conductor travelling with you, and ready on the boat or at any of the changes to advise or help, and in 24 hours you find yourself landed in some big, roomy Swiss hotel, right up amongst the eternal snows! However tired you are, you look upon these hills, and peace and rest descend upon you. By this time you have made friends at table or in the train with those of the party who are most attractive to you, and with them you proceed to make venturesome journeys across glaciers or attempt to scale some alpine height. The flowers are a perpetual feast of colour; the wind amongst the pines is a perpetual feast of song. In the evenings, less ethereal enjoyments are indulged in, for the old maids always insist on getting up a dance, and the old bachelors arrange progressive whist parties. Sometimes charades and dumb-crambo end up the day.

Though you have only paid for a week or 10 days in the hotel, or hotels, your return ticket lasts three weeks, and you are free to make any further plans and return at your leisure. Others of the party are certain to be extending their holiday to a fortnight or more, and having become familiar with the ways of Swiss hotels, and with the few necessary phrases of French or German, you gaily join forces with one or more companions and go off to some other vantage point—perhaps to some rough little châlet still higher up in the Alps, which you have visited by walking from your hotel below. Is it very mundane to suggest that the omelettes, and the veal cutlets, at these little châlets form a delicious contrast to the usual hospital meals?

The days fly by; holidays always go fast when they have taken long to earn; and all too soon, with brown cheeks and cheery voice you are back in the ward again at the old round of work. But how easy it all seems! The vigour and joy still pervades all your person, you feel as though nothing could tire you or your patience. And after three weeks without mentioning hospital affairs how interesting it all seems! The moral is that the open-air life is the one which renews the mind and body, and that it is a good thing for the eyes to behold the sun. It is sad that we should have to live so much amidst darkness and sorrow, but at least let us fly to the mountains and the sunshine whenever we have the chance.

Of course, it is not necessary that, in order to enjoy a holiday, it should be spent in Switzerland. The United Kingdom and Ireland offer a very wide field of choice, sufficient to suit the tastes of all sorts of nurses. For those who prefer a bracing air there are pleasant places on the Norfolk or Yorkshire coast; Devonshire and Cornwall have charms for those who prefer a milder climate; there are the hills of North Wales; the beautiful canals and moors of Scotland; and the lakes of Cumberland and the sister island, not to speak of the numerous more or less attractive resorts in closer proximity to the metropolis. Given the chance of a holiday and of a few pounds to spend, there can be little difficulty in deciding where to go.

### The Modern Nursing of Consumption.

By Dr. JANE H. WALKER, Physician to the New Hospital for Women, London, and Medical Superintendent of the East Anglian Sanatorium, Nayland, Suffolk.

### LECTURE IX.

### DISINFECTING.

*Disinfectants.*—There is a very large number of disinfecting agents on the market at present, most of them of doubtful efficiency. Everyone has probably their own peculiar fancy; we can only mention one or two here.

*Perchloride of Mercury* is of course one of the strongest. It has the advantage of having no smell. On the other hand this absence of smell makes it rather a dangerous drug to have about in a private house. A nurse should not use it, nor have it in her possession, without the doctor's orders.

*Carbolic Acid.*—The drawbacks to this common disinfectant are that it smells nasty, is dangerous to leave about, easily causes burns, and, from a disinfecting point of view, it is said to coagulate the albumin in sputum so rapidly that the bacilli may get enclosed in envelopes of coagulated albumin and so escape destruction. The usual strength for this disinfectant is ½ to 1 ounce to a pint of water.

*Jeyes Fluid* is used by many. The smell permeates the whole house, and is much objected to by most people. Of its efficacy we have made no trial.

*Chloros.*—This is said to be extremely powerful as a disinfectant; it has a very faint and not peculiarly disagreeable smell, does not burn the hands or clothes, and on the whole we find it as good as any other antiseptic, and much better than many which we have tried. It is really sodium hypochlorite. It can be obtained from the United Alkali Company, of Birmingham. The usual strength to use is 1 in 80. It can be used to put into patients' sputum cups, to wash out the sputum flasks, bedroom utensils, and to swab down the walls of the rooms (provided, of course, that the wall covering be washable).

*Sputum.*—In a sanatorium this should be mixed with sawdust and burnt in the furnace. In a private house it should be mixed with whatever disinfectant be used, and emptied down the W.C.

*Utensils.*—These should be washed out with very hot water and chloros, or, if entirely of china, hyd. perchlor (1 in 1,000) may be used.

*Rooms.*—If Hall's, or some other washable distemper be used for the walls, they can be rubbed down with chloros (1 in 80), or sprayed with formalin. If the room is to be entirely disinfected, the doors and windows should be closed and papered up, and a sufficient number of tabloids of formalin should be burned in a formalin lamp. Full directions are given with the formalin lamps, which may be got from any good chemist.

*Linen Handkerchiefs* should be steeped in 1 in 80 chloros, or 1 in 20 carbolic, before being sent to the laundry. Some people advocate using Japanese handkerchiefs, which can, of course, be burned after use. As a matter of fact they are not of the smallest use so far as purpose of blowing the nose goes. Aseptic dental handkerchiefs are better, and should be used for all very bad cases, where there is difficulty in keeping the mouth clean. Like the Japanese fibre ones they can be easily burned after use.

*Table napkins* should be boiled or soaked like the handkerchiefs before going to the laundry. They should be kept in linen cases (made like night-gown cases on a small scale) which can be washed weekly.

*Sputum Cups.*—Plain mugs with metal covers, after the pattern of those used at Nordrach, are the best. They can be obtained cheaply at Wilson's in Wardour Street.

*Flasks.*—Pocket flasks are of course a necessity for all patients who are going about. The best are Dettweiler's, or a modification of his. They are made on the principle of the non-spilling office ink bottles, and can be carried in any position. Cheaper kinds are apt to break, or the lids to leak.

In the case of poor patients the very great importance of always carrying one of these flasks must be pointed out, and if possible they must have thoroughly impressed upon them the fact that even at home a cup or flask is to be used and not the kitchen fire.

### NURSING AMONG THE POOR.

We feel that it may be said the treatment depends so largely upon food, rest and pure air, what can be done by the poor in their own houses? If a poor patient has ever been in a sanatorium he can very generally be depended upon to keep the windows open, and feed up as far as possible. But there are so very many more poor patients in every town than can be provided for by the cheap sanatoria at present available, that there must always be hundreds of cases in all stages of the disease to be treated and nursed in their own homes. As a rule, these patients go weekly or fortnightly to the out-patients' department of some hospital, there to get cod-liver oil, creosote, and perhaps a perfunctory command to sleep with their windows open. They live in a questionably clean room, a room generally shared by several other people, the food is of the least nutritious, most unwholesome kind, and often largely supplemented by "ale" and spirits to keep up the strength. They wonder that they are sick, and that they cough and sweat at night. The worse they get the more dirty flannel and flannelette garments they put on, the less they wash and the less they eat. When they are quite bedridden a doctor or district visitor sends the district nurse, who can do little beyond washing the patient and administering the inevitable cough mixture.

Three things are really needed. Firstly, time on the part of the overworked out-patient physician to enable him to fully explain to patients the imperative need of fresh air, cleanliness and food. Secondly a liberal distribution of handbills to out-patients, setting forth the evils of spitting, of stuffy rooms, and of over-clothing; giving also simple directions as to diet. Quite cheap food can be nutritious, the 4d. spent on beer daily, the weekly money for tobacco can provide milk, potatoes and suet. A few homely receipts for suet puddings, Scotch broth, milk puddings, dumplings, etc., might well be included. Excellent handbills and small pamphlets are published by the National Society for the Prevention of Tuberculosis, and they should be more widely distributed than is done at present by doctors, nurses and clergy.

Thirdly, district nurses should be conversant with the rationale of the so-called "open-air treatment." Even in the slums much can be done by open windows, soap and water and washing, to keep both the house and the patient healthy.

All cases of phthisis should be reported to the nurses, of whom there are still far too few, and they might give a weekly, or even fortnightly visit, to see that the patient and his relations are living as hygienically as circumstances permit. She could give them hints as to food, clothing, exercise, and rest. The advantages of a drive on the outside of a 'bus or tram are not sufficiently appreciated by our poor. It gives change of air and surroundings without much fatigue, and many women and children of the poorer classes suffer much from being always in the atmosphere of a few small streets, and rarely getting away from a mile radius of their own homes. When they do take a 'bus "ride" they go inside.

The nurse can impress on the poor the fact that damp air or rain will not give them their "deaths of cold," nor having the windows open in all weathers increase their coughs.

Of the cases which are bedridden we can say nothing.

The average district nurse is beyond praise in her nursing of the poor. The only thing we can beg her to do is to see that the patients have some sort of sputum cups and that as much fresh air as possible is admitted to the room.

Below we give a weekly diary which may be useful in nursing private cases. By this the doctor can see at a glance how the patient has got on between his visits, and can add his own notes as to the variations in the course of the disease.

| Name. | Date. | Lungs. | Temp. | Pulse. | Drugs. | Diet. | Exercise. | Weight. | Condition during Week. |
|---|---|---|---|---|---|---|---|---|---|
| | | | | | | | | st. lbs. | |
| Smith, Henry. Age 25. | Aug. 1-8 | — | 99·1-100·5 | 88-96 | None | Ordinary | Half mile. A.M. Garden. P.M. | 6 0½ | Good |
| | 8-15 | Slightly increased crepitation R. base | 100-102 | 100-102 | — | Ordinary | Bed since Wednesday | 6 2½ | Slight hæmorrhage Wednesday. None since |
| | 15-22 | — | 100-99·8 | 90-88 | None | Ordinary | Bed | 6 3 | No hæmorrhage. Much the same |
| Brown, Emma. Age 21. | Aug. 1-8 | Pleurisy L. lung .. Friction L. ax; no effusion | 99-103·5 | 100-108 | Strapped | Ordinary | Bed | 7 5 | Rise of temperature Thursday Pain L. side all Thursday and Friday |
| | 8-15 | Slight friction L. ax. still | 100-99·5 | 108-98 | Still strapped | Ordinary | Bed | 7 4½ | Better. No pain |
| | 15-22 | No friction .. .. | 100-98·5 | 90-88 | — Salts Thursday morning | Ordinary | Bed till Thursday. Up on couch since then. P.M. | 7 6 | Better. No pain. Bad headache Wednesday. Sick Wednesday evening. Not since |

### How to Become a Midwife: The Rules of the new Act Explained.

By E. L. C. Appel, M.B., B.S., B.Sc.(Lond.).

*Continued from page 194.*

*General* (continued).

*Clause* 18.—Notification.

1. *Deaths.*—In all cases in which the death of the mother or of the child occurs before the attendance of a registered medical practitioner the midwife shall, as soon as possible after the death, notify the same to the local supervising authority.

2. *Stillbirth.*—In all cases where a registered medical practitioner is not in attendance the midwife shall, as soon as possible after the occurrence of a stillbirth, notify the same to the local supervising authority.

A child is deemed to be stillborn when it has not breathed or shown any sign of life after being completely born.

3. *Puerperal Fever and other Infectious Diseases.*—These cases are included in the notice required when medical help is sent for. (See Clause 19 (*b*) below.)

*Clause* 19.—A midwife shall keep the following records:—

(*a*) A register of cases in the following form:—

No. ........

Date of engagement to attend ..........................................

Name and address ..........................................

No. of previous labours and miscarriages ..............

Age ........

Date and hour of midwife's arrival ..........................

Presentation ..........................................

Duration of 1st, 2nd, 3rd stage of labour..........................

Complications (if any) during or after labour...........

..........................................

Sex of infant ...........Born living or dead ...............

Full time or premature.—No. of months ...............

If doctor called...........Name of doctor.................

Date of midwife's last visit..........................................

Condition of mother then (*see Clause* 11 above).

..........................................

Condition of child then ..........................................

Remarks * ..........................................

..........................................

* If any drugs have been administered, state here their nature and dose, and the time and purpose of their administration.

(*b*) A record of sending for medical help, in the following form:—

No................ Date..............................
Name of patient ..........................................
Address ..........................................
Requires medical assistance at once on account of ...
..........................................
Signed ........................ (Certified Midwife)
Sent to (doctor) ..........................................
At (address) ..........................................
Time of sending message ..........................................

The midwife shall make two copies of the above (*b*) by means of transfer paper or otherwise; she shall preserve one of these copies for herself, and shall send the other by post to the local supervising authority within twelve hours (*see clause* 18 (3) above).

*The midwife is also recommended to keep a case-book with fuller details.*

*Clause* 20.—The supervising authority shall make arrangements to secure a proper inspection of every midwife's case-book, bag of appliances, etc., and, when thought necessary, an inspection of her place of residence, and an investigation of her mode of practice.

*Clause* 21.—Nothing in this section (*i.e.* in the foregoing twenty clauses) shall apply to certified midwives exercising their calling in hospitals, workhouses, or Poor-law infirmaries under the supervision of a duly appointed medical officer.

*Case-book.*

Clause 19 of the regulations requires the midwife to keep a record of her cases, with particulars of each case.

Case-books for this purpose can be obtained, price 1s. each, by post 1s. 5d., from The Scientific Press, Ltd, 28 & 29 Southampton Street, Strand, W.C.

An inspection of the midwife's case-book may at any time be ordered by the local supervising authority.

*Forms for Sending for Medical Help.*

Books of forms for sending for the doctor can be obtained, price 6d., by post 7½d., from The Scientific Press,

Ltd.[9] Clause 19 of the regulations also requires the midwife to keep a record of sending for medical help.

A copy of this record must be sent by post to the local supervising authority within 12 hours after the midwife has sent for medical help.[10]

*Employing a Substitute.*

A certified midwife is not allowed to employ an uncertified midwife as her substitute.

*Emergency Cases.*

A woman, though not certified under the Midwives Act, may render assistance to a lying-in woman in case of emergency.

*Certificates of Death, Stillbirth, etc.*

A woman certified under the Midwives Act is not allowed to give any medical certificate, or any certificate of death or of stillbirth.

A medical certificate, if required, must be obtained from a doctor.

If a death or stillbirth has occurred in a midwife's practice, and no doctor was present at the case, the midwife must report the death or stillbirth, as soon as possible after its occurrence, to the local supervising authority.[11]

*Abnormalties in Childbirth, and Disease.*

A woman certified under the Midwives Act is not allowed to undertake cases of abnormality or of disease in connection with childbirth. She may only undertake the charge of cases of normal labour and of lying-in. In all cases of abnormality or of disease a doctor should be sent for.

[9] All Midwife Forms (price 1d. each, postage extra) can be obtained from The Scientific Press, Ltd., 28 and 29 Southampton Street, Strand, London, W.C.
[10] See Clause 19 (*b*), above. [11] See Clause 18, above.

[These articles are published in book form by the Scientific Press, Ltd., 28 and 29 Southampton Street, Strand, W.C., at 1s., and 1s. 1d. post free. All midwife forms, price 1d. each, postage ½d. extra, can also be obtained from the Scientific Press.]

(*To be continued.*)

### The Central Midwives' Board and the Examination Scheme.

A MEETING of the Central Midwives' Board was held at the offices on Thursday last week. There were present:—The Chairman (Dr. Champneys), Mr. J. Ward Cousins, Dr. Cullingworth, Miss Oldham, Miss Paget, Dr. Sinclair, Miss Wilson, and Mr. E. Parker Young.

The first business was the consideration of an examination scheme, drafted by Dr. Cullingworth, amalgamating in his view the best points of two schemes which had previously been reported upon by a Standing Committee. The clauses were taken in order and voted upon. It will be remembered by those who read Dr. Sinclair's recent letter to the medical Press that a difference of opinion had arisen between members of the Board with regard to the appointment of examiners, the Standing Committee having recommended "that one or more women who hold, or have held, the post of matron of a lying-in hospital, or midwife, if otherwise qualified," might be appointed examiners.

This question at once came to the fore, and a resolution was moved by Mr. Ward Cousins to the effect that members of the medical profession only should be eligible for appointment as examiners. He considered that every detail of the training and examination of a midwife would be more fitly dealt with by medical practitioners, including such matters as infant feeding, sanitation, and nursing.

Mr. Parker Young took the same view, saying that in his opinion nursing could only properly be taught by medical practitioners.

Miss Wilson thought that the hands of the Board should be left free in the matter. It was desired to make midwives good nurses as well as good midwives; the Board demanded nursing experience, and it was reasonable that examination in nursing should also be required.

Dr. Cullingworth considered that as the appointment of examiners was entirely in the discretion of the Board, they should be free to appoint whom they would. Most of them knew women competent for the work. Dr. Sinclair held, on the contrary, that there should be a declaration of policy on the question, instead of leaving the Board "free" to deal with it when two or three members only were present.

After some further discussion, in the course of which Mr. Parker Young asked "if the Board could really seriously contemplate asking medical examiners to sit at the same table and examine with a midwife," and Dr. Sinclair threw in a remark to the effect that the next thing would be a "highly gifted chambermaid," the resolution was put. As only Dr. Sinclair and Mr. Parker Young supported it, the rest of the Board voting against its adoption, it was lost.

It was agreed that "application for the post of examiner be invited by advertisement;" that no examiner be allowed to examine his or her own pupils; and that every examination, written and oral, be conducted by not less than two examiners. Another discussion ensued with reference to the number of examinations to be held every year, the scheme providing for two only, to be held in London and provincial centres simultaneously. Miss Paget pointed out that so long an interval would be hard upon those who failed to satisfy the examiners, and could not come up again for six months, during which time they would be unable to practise, while their services might be much needed. A resolution in favour of three examinations a year was lost, all the medical members voting against it, and an amendment proposed by Mr. Parker Young, putting in the words "oftener if necessary" was carried. Other clauses were carried without discussion or postponed for consideration at an adjourned meeting, to be held on July 14th.

After consideration of applications for certificates, the names of 829 women were passed under Section 2 of the Act, and ordered for entry on the roll. The total number now enrolled is 6,159.

Letters were read from the secretary of the Rotunda Hospital, Dublin, acknowledging the receipt of a copy of the resolution passed by the Board on May 26th, varying the terms of Form IV. in the schedule to the rules, and stating that the matter had been referred to a special meeting of the governors of the hospital. A letter was read from Dr. J. W. Byers, of Belfast, acknowledging the receipt of a copy of the same resolution, and stating that the Incorporated Belfast Maternity Hospital will be satisfied with the proposed reduction of the puerperium from ten to eight days. Other letters were dealt with, including one from Dr. McLean, of Cardiff, announcing courses of instruction in midwifery, and that the local branch of the Queen Victoria's Jubilee Institute had undertaken to add a maternity department to their work; and another from a Portsmouth medical officer of health calling the attention of the board to a midwife who appended the letters R.C.M.B. after her name. The Board agreed that a midwife was only entitled to describe herself as "certified midwife" in full.

After disposing of financial and other business, the Board proceeded to consider applications from institutions for recognition of certificate, etc., and from medical practitioners as teachers and midwives for signing Form III., and Miss Paget called attention to the fact that private and personal matters relating to individuals, usually dealt with by standing committee, would come up now that the Press were present, and she thought it hardly fair to discuss them in public. A resolution to ask the Press to withdraw was, however, not carried.

The following institutions were approved as institutions for the training of midwives under Section C of the rules, and their certificate recognised:—Bedford Row Lying-in Hospital, Limerick; the Eden Hospital, Calcutta.

The following institutions were also approved as training schools:—The Salvation Army Maternity Hospital, and the Bristol General Hospital. Dr. Sinclair moved the rejection of the Salvation Army Hospital on the ground that the Salvation Army is an autocracy, and therefore necessarily inefficient in its organisations; it was sufficient, in his opinion, to know a medical man to be a member of the Salvation Army to prove him unfit for his profession. This reasoning was not followed by the rest of the Board, and no one seconded or supported the resolution.

The rest of the business on the agenda was postponed, the board having sat from 2 45 to 5.40 P.M.

### Drawing-room Meeting for Nurses.

ADDRESS BY SIR WILLIAM COLLINS.

By the invitation of Lord and Lady Portsmouth a large number of nurses were entertained in their beautiful new house, 16 Mansfield Street, W., on Friday last.

The meeting was arranged by Miss Hilda Dillon, Hon. Secretary and Treasurer of the Nurses' National Total Abstinence League, who, with Lady Portsmouth, received the guests at half-past three. Among those present were the Hon. Mrs. Eliot Yorke (President of the League), Lady Biddulph of Ledbury, Mrs. T. P. Whittaker, Miss M. E. Docwra, and Miss Orme, vice-presidents; Miss Richardson, Matron of the Temperance Hospital, and many others.

At four o'clock Lady Portsmouth welcomed the large assembly and introduced Sir William Collins, M.D., who gave a most interesting and instructive address.

At the beginning of his address Sir William Collins said that as a jaded surgeon, and weary county councillor, he could not help feeling that some other than he would have been better to address the meeting, and he suggested a bishop, a poet, or a politician. He then referred to the development of women's work during the Victorian Era, alluding particularly to nursing. He spoke of the natural dissatisfaction a woman has to work which does not appeal to the heart as well as to the head; and he thought that, in a nutshell, the satisfaction of nursing could be thus explained. On looking back, he considered that nursing had been developed by three impulses—religion, war, and science. The last impulse claims the highest knowledge. There might be less heroism now, than formerly, attending the work, but he thought that nursing in some of the slums of London required as much heroism as attending the wounded on a field of battle. With regard to the temperance movement, he felt that a bond such as temperance reform was a grand mutual interest among nurses. In supporting temperance work there was a counter-current of interest which was of great social service. A hospital was a school in which only one thing was taught, and nurses should use some of their scanty leisure in studying some social reform. He believed that some central institute, some training college, giving nurses an opportunity to specialise, would be excellent, as there seemed to be too many nurses of the same pattern. He was of opinion that a greater variety of aspiration was needed; something beyond the desire to nurse a great surgical case.

Returning to the temperance question, Sir William expressed strong views against quacks and secret cures for inebriety, and appealed for a rational and open-handed dealing of the question. He challenged any quack to produce a cure out of a bottle, and believed that the reason for secrecy was the financial reward brought by the halo of secrecy. No one should proffer a cure unless he was willing to make such a cure public. During the 16 years he had been surgeon at the London Temperance Hospital, he had always found that abstainers were the best patients to operate upon. He sincerely hoped that the nucleus of temperance nurses would lead to a greater extension of social reform.

The President of the League then expressed her thanks to Sir William Collins for his address. Thanks were also voted to Lady Portsmouth for allowing the meeting to take place in her house.

Lady Portsmouth, in reply, said that it had given Lord Portsmouth and herself great pleasure to welcome the nurses and that it was the first large meeting that had been held in their new house. She invited all those present to tea and to remain as long as they could to the excellent concert which had been begun before Sir William Collins's address.

### Appointments.

CAMPBELL HOSPITAL, PORTROY, BANFF.—Miss Margaret J. Barles has been appointed matron. She was trained at the Glasgow Children's Hospital and Radcliffe Infirmary, Oxford. She has since been matron of the Fever Hospital, Peebles, N.B.

CHESTERFIELD INFIRMARY.—Miss B. McCurdy has been appointed night sister. She was trained at the Birkenhead Infirmary, where she has since been ward sister, theatre sister, and night sister.

HOLBORN UNION INFIRMARY.—Miss Annie T. Hyde has been appointed superintendent nurse. She was trained at Southwark Union Infirmary and has since been assistant nurse at the Ophthalmic School at Hanwell.

MANCHESTER CHILDREN'S HOSPITAL.—Miss Nora Woodhouse has been appointed sister. She was trained at the Infirmary, Sunderland.

PLYMOUTH WORKHOUSE HOSPITAL.—Mrs. M. Williams and Miss A. N. Houlding have been appointed charge nurses. Mrs. Williams was trained at Queen Charlotte's Hospital, London, and the West Herts Infirmary, Hemel Hempstead. She has since been on the private staff of the Royal Berks Hospital, Reading. Miss Houlding was trained at Chorlton Union Infirmary, West Didsbury, Manchester.

SOUTHWARK UNION, LONDON.—Miss Sarah H. P. Leslie has been appointed superintendent nurse. She was trained at St. Bartholomew's Hospital, London, and has since held appointments in the Indian Nursing Service, both civil and military.

WARRINGTON INFIRMARY.—Miss L. Crowther and Miss C. Hammond have been appointed charge nurses. Miss Crowther was trained at the North Staffordshire Infirmary, and Miss Hammond at Chester Infirmary.

### "The Hospital" Convalescent Fund.

THE Hon. Secretary begs to acknowledge with thanks the receipt of 20s. from a nurse who wishes to remain anonymous, 5s. annual subscription from Miss Simmons, and 2s. 6d. per the Travel Correspondent of THE HOSPITAL.

### Everybody's Opinion.

### THE RURAL MIDWIVES' ASSOCIATION.

Mrs. Heywood Johnstone writes: Instances having come to our knowledge of applications for help from various bodies, for the training of midwives, under names and on lines similar to those used by the Rural Midwives' Association, my committee would be grateful if you would kindly insert this letter to say that no application for assistance or co-operation to the work of our Association is authentic unless signed by our secretary, Miss Stutchbury, Office Rural Midwives' Association, 47 Victoria Street, S.W., or by myself, as Chairman of the Executive Committee. I may here add that our Association is first in the field of active work, having in the last 12 months trained and sent a considerable number of cottage midwives to different counties.

### A WARNING TO MATRONS OF NURSING INSTITUTES.

"The Matron of the Bootle Institute for Trained Nurses" writes: I wish to thank you for your note on my letter in your issue of June 25. The plan for preventing such losses which you were good enough to suggest is scarcely possible, as people who are honourable in their payments would naturally be offended at the implied suspicion; but to obviate the loss of a large amount the account might be sent in weekly to persons suspected of shirking payment, and if that is not settled in reasonable time to withdraw nurse from case. I have profited by my recent experiences, and I feel sure that your note will be a warning to other matrons who may be similarly situated.

### A QUESTION OF RELIGION.

"An Irish Protestant who likes justice" writes: I have just read "Convert's" letter, in which she complains that Roman Catholic nurses cannot easily get appointments on account of their religion, and I feel that out of justice to Protestants her letter should be answered. If "Convert" is an Englishwoman, no doubt she is not aware that the Protestant nurses, hailing from the South and West of Ireland, who join the Queen Victoria Jubilee Institute are not allowed by the Roman Catholic priests to work in their own counties, and have, in consequence, to be sent to Ulster or to Great Britain. The priests would order the people to boycott a Protestant Queen's nurse in the South. If "Convert" thinks that Protestant England is unjust to Roman Catholics, in which view I think she is greatly mistaken, she certainly should be made to understand what disabilities we Protestants are under in the Roman Catholic parts of Ireland.

### RED TAPE IN THE WARDS.

"A London Matron" writes:—It is with much pain I have read "Red Tape in the Wards" in your paper. Such people as the writer describes do exist, and in 16 years of hospital life I have met them—to the number that I could count on the fingers of one hand and leave some over! I think of the sisters of wards in which I have worked, their tender womanliness and upright dealings to patients and ward; and hard-worked probationers alike in our London hospitals; and my heart burns that such stories should be raked up and placed before the public as usual occurrences. Too clean nurses, too tidy wards, too dirty feeding-cups, dirty tricks with unwashed medicine cups, active bad nursing of helpless cases, surely the paradox here presented to thoughtful minds shows the happy ignorance of the writer of what our wards really are. Let the patient—who "dare not complain"—answer for himself as he smiles happily to see his nurse back on duty after her all-too-short rest. "Off duty, miss?" he says; "the very sight of yer bonny face, let alone yer kindly ways, does a feller more good than all the doctor's stuff!" This was said in a big London hospital ward, two months ago, to a young woman, who was learning to "lose her heart" in her work—aye, and lose it gladly like countless others, "blessed," as says Carlyle, "in having found her work." Thanking you for allowing me to trespass on your space to make a protest echoing in many hearts to-day.

### Novelties for Nurses.

By Our Shopping Correspondent.

### EIFFEL TOWER JELLIES.

These jellies are prepared in the solid block form which has proved so popular. They are very nicely flavoured with lemon, orange, and other fruit flavours. The idea that home-made jellies are more nutritious has passed away, and with it much inconvenience and vexation of spirit, for jelly-making is a messy and by no means easy process. Moreover, some days' preparation was often necessary. Now, thanks to such preparations as the Eiffel Jellies, and the use of a little ice, in a very short time the invalid may have before him a clear and firm jelly, containing all the pleasant and stimulating qualities which render such food useful in the sick-room. The district nurse well knows the value of so cheap and tempting a commodity which is obtainable everywhere.

### Where to Go.

Evening fête in aid of the East London Hospital for Children, Shadwell, at the Royal Botanical Gardens, Regent's Park. Tickets, 25s., 15s., and 5s. Friday, July 15, 10 p.m.

### TRAVEL NOTES AND QUERIES.

By our Travel Correspondent.

Holidays in Ilfracombe (Mrs. L.). Owing to a printer's error, the word "inexperience" was put for "inexpensive," and "Axford" for "Oxford." If you did not understand, write to me again.

Addresses Wanted in Bruges, etc. (E. Y. B.). I will send you the addresses with pleasure when I know what you want and what you can comfortably afford to pay. What is suitable for one correspondent is often wholly unsuitable for another, so let me know something of your requirements, and especially what you can spend. The cheapest terms I know of in Candebec are 7 fcs. per day. It is a charming spot.

Lodgings in St. Malo (Chislehurst). I hope this will catch your eye, but you give me no pseudonym. No, there is no charge made for answers in this column. What you ask for does not exist in our sense of the word upon the Continent, and if it did, £1 a week is too little to have really good food. Such a sum, you see, means only 3s. a day for board and lodging. August is the most expensive month in all the year in which to seek accommodation. Are you tied to St. Malo? The old city is not agreeable in summer because it is enclosed with high ramparts. Moreover, living is dear there. I could tell you of cheap places at St. Servan, close by, but nothing at your terms. Write me again more fully of your requirements. I could give you addresses in Belgium in nice places for very low terms, and the journey would not be so expensive. If you write to me directly you see this I can reply in the following issue.

Holidays at Aberdovey (A. B.). I am afraid that nothing in the way of accommodation is cheap in August; every room is full, and proprietors can ask and get what they choose. We never give addresses of rooms, only of hotels, pensions, and occasionally of ladies who take paying guests. Aberdovey is a quiet, rather dull place, but with fresh air moderately bracing. There are some good walks round, where really fine scenery is to be found—notably, Llyn Barfog, the "Happy Valley," and the lovely Llyfnant Valley—reached by taking train to Glandovey. There are two hotels, the "Dovey" and the "Marine." You might go to one of these for the night and look for rooms. I was told the other day of a Mrs. Richards who has rooms, and also takes boarders very reasonably. Her address is 3 Bodfor Terrace. I know nothing of her personally. There is fairly good bathing, but the coast is not interesting. Would you care for the Isle of Man? It would not be so expensive, and, to my mind, it is more interesting. I could give you a reliable address of very reasonable quarters there in Douglas. Let me hear again if I can help further.

### Echoes from the Outside World.

### Movements of Royalty.

The King returned to London on Friday evening, and was met at Charing Cross Station by the Marquis of Lansdowne, with whom he had some minutes' conversation before leaving. Subsequently his Majesty drove to Buckingham Palace, being loudly cheered by the assembled spectators as the carriage passed out of the station. On Saturday afternoon the King left London on a visit to Lord and Lady Gerard at Eastwell Park, near Ashford. In honour of his visit the huge crown cut in the Kentish Down in the vicinity of Ashford by students of Wye Agricultural College, as a memorial of his Majesty's Coronation, was brilliantly illuminated on Saturday night. On Monday the King, after his return to London, formally received from the Australian Commonwealth the historical picture representing the opening on May 9, 1901, by the Prince of Wales of the first Parliament, which is at present exhibited at the Academy by Royal command. The ceremony took place in the Royal Academy, and his Majesty was accompanied by the Queen and the Prince and Princess of Wales. The King said that he accepted the picture with great satisfaction as the souvenir of a most important event in the history of the Empire.

The Queen paid a private visit on Thursday last week to the Docks at the East End, and, at her own request, viewed the ivory floor of one of the great warehouses at the London Docks as it ordinarily appears. The warehouse workmen were not therefore called away from their employment. An acre of ivory tusks of all sizes, and from all countries where ivory is produced, covered the floor. The Queen spent nearly an hour in the warehouses, and afterwards drove to the dock warehouses in Cutler Street. Here she inspected a great stock of Turkish and Persian carpets, a collection of ostrich feathers, and also of feathers of various birds whose plumage is used for decorative purposes. Her Majesty was likewise shown silks of fine texture from India and China and a number of Japanese and Chinese curiosities.

### Progress of the War.

Official accounts of the battle of Fen-shui Ling show that the Russians had prepared a position of such strength that it could not have been taken by direct assault without an enormous sacrifice of life. Ninety bodies of Russians were found on the main road, and six officers and 82 men were made prisoners. After the battle the Russians attempted to recapture the position with three battalions and 16 guns, but ultimately they were finally driven back. The Japanese are stated to have landed an independent division of 10,000 men on June 24 at the naval base on the Elliot Islands, making 180,000 men in the field. According to a telegram from General Kuroki's headquarters, the Russians have retreated all along the line before the northern advance of the Japanese, and the country is now almost clear to the north-west. An official Japanese message states that the reports of atrocities committed by the Japanese are entirely without foundation.

### Death of Mr. Watts, R.A.

The most honoured of English artists died on Friday in the person of Mr. G. F. Watts, R.A. Notwithstanding his age, Mr. Watts continued painting until he was attacked by bronchitis last month, and he passed quite peacefully away at his residence in Melbury Road, Kensington. A native of London, he was born on February 23rd, 1817, and early in life showed a taste for art. When he was 20 he exhibited at the Royal Academy two portraits of ladies and a picture of a wounded hero. Five years later he obtained a prize of £300 for his design of "Caractacus being led in triumph through the streets of Rome," and four years later a prize of £500 for his "Alfred inciting the Saxons to resist the landing of the Danes." He was also commissioned by the Government to paint a fresco of "St. George and the Dragon," which was completed in 1859. His works were symbolical, classical, and romantic, and he treated a few modern subjects. He was an able portrait painter and a masterly sculptor. The "Watts Collection," which represent the greater part of his life work, is now housed in the Tate Gallery. Mr. Watts twice declined to accept a baronetcy, and the only distinction he consented to receive was the Order of Merit from King Edward. His favourite flower was the daisy, because, as he used to say, "though a humble thing it looks up." At his own desire his remains were cremated.

### Wreck of an Emigrant Ship

Tidings reached England on Monday of a terrible disaster which had befallen a Danish emigrant ship, the *Norge*, in the Atlantic on Tuesday of last week. The news was brought to Grimsby by a steam trawler which had on board 27 of the survivors. The following day two more parties were landed at Stornoway, including the captain, who had gone down with his vessel, severely injuring his leg in the wreckage, but after swimming about for nearly two hours was rescued by one of the lifeboats which had been launched before the ship sank. Although the shipwrecked persons, scantily clothed, had been six days at sea and the only provisions on board were two casks of fresh water and a box of bread, the only death had been that of a little child who died on Saturday morning and was buried at sea. The captain states that of the 13 women in the boat "two rendered noble assistance in cheering up the rest." The mate of the *Norge*, finding himself in a boat which was dangerously full, jumped into the sea to swim to another where there was room but no one to take the head. But he perished in the attempt. It is feared that over 600 lives have been lost.

### The Admission of Women to Dublin University.

On Thursday last week a number of women were for the first time participators in the degree-giving ceremony at Trinity College, Dublin. Next session the College opens its doors to the fair sex, and this step has been celebrated by the bestowal of honorary degrees upon three distinguished Irishwomen, namely, Mrs. Bryant, head mistress of the North London College, and Miss Jane Barlow, the well-known writer, who received the degree of Doctor of Literature; and Miss Mulvany, mistress of the girls' school in connection with the Alexandra College, Dublin, who received that of Doctor of Laws. Six former students of Girton and Newnham, who had passed examinations qualifying for an honours degree at Cambridge, presented themselves for the Dublin B.A. degree.

### An M.P. Killed in a Motor-car Accident.

A motor car accident, by which Sir William Rattigan, K.C., M.P. for North-East Lanark, lost his life, occurred on Monday afternoon. Sir William, with Lady Rattigan and their chauffeur, were on their way from London to Scotland, in a Darracq car, and were approaching a village near Biggleswade, at a speed of 10 miles an hour, when the spokes of the near hind wheel scattered as the car was rounding a corner. The car overturned, Lady Rattigan was thrown over the chauffeur in a heap, and Sir William was dashed against the front seat, and fell with his head partly hanging over the side of the back compartment. A cyclist and some labourers working near rendered assistance, and Sir William was found to be dead; Lady Rattigan was badly cut by broken glass, and the driver escaped with comparatively slight injuries.

### Notes and Queries.

**FOR REGULATIONS SEE PAGE 143.**

*Hospital Training.*

(105) Can you tell me of any good nursing homes at Brighton where young probationers are received?—*A. B.*

The Royal Alexandra Hospital for Sick Children, Dyke Road, takes probationers of 20. Premium £20.

*Home.*

(106) I should be much obliged if you could tell me where a nurse could be received for Weir-Mitchell treatment. She is not able to pay the usual fees.—*E. M. M.*

There is no public institution for the treatment, but private institutions frequently advertise it. A medical man would advise you.

Is there a home, preferably at the sea, which would take a boy of 10, who suffers from epileptic fits, for three weeks or so? Parents could pay 10s. a week towards cost.—*G. H.*

Possibly the Secretary, the National Society for Employment of Epileptics, 12 Buckingham Street, Strand, W.C., could help you.

Can you tell me where, for a small payment, a girl aged 13, who suffers from epileptic fits, could be received?—*No Name.*

See reply to *G. H.* The Meath Home of Comfort, Godalming, might be suitable.

Will you kindly tell me where a helpless young man of the middle class could be received? His friends could pay a small fee.—*M. W.*

See "Burdett's Hospitals and Charities." You give too few particulars to enable us to select a possible home.

Can you kindly tell me the names of any homes or schools where a boy of eight, suffering from mental weakness, could be received? His relatives could pay for him.—*M. C.*

Write full particulars to the Secretary, the National Association for Promoting the Welfare of the Feeble-Minded, 53 Victoria Street, S.W.

*Bicycles.*

(107) Can you tell me of any cycle shop or agency where bicycles can be hired by nurses for an hour or two, and what charge is made?—*M. F. R.*

Most cycle shops hire out cycles by the hour. The cost is usually 1s. for the first hour and 6d. per hour after.

*Australian Trained.*

(108) I enclose stamped envelope wishing for an early reply. Would a fully-qualified nurse, trained in a good hospital in Australia, be eligible for posts in English hospitals without furthe training?—*K. S.*

A query requiring a private reply should always be accompanied by a fee of 2s. 6d. Yes, but the best training schools are overwhelmed with applications for vacant posts and mostly promote their own nurses.

*Stammering.*

(109) Where could a boy of 16 be cured of stammering? The parents could pay a small fee.—*B. J.*

Try a course of elocution lessons. They may be obtained at the Polytechnic institutions very cheaply.

*Inscription.*

(110) I am anxious to present a doctor with a piece of silver plate in gratitude for private tuition for the L.O.S. Will you kindly tell me how to word the address, and give me the names of some silversmiths in London where I could buy the plate?—*A. B.*

For names of silversmiths see advertisements in any London paper. We cannot suggest a suitable inscription, knowing so little of the circumstances; but if you send particulars we will help if you wish. Have you no literary friend whom you could ask?

*Address.*

(111) Is Dr. Henry G. Spooner, who wrote the article in The Hospital for October 31st, 1903, a specialist for diseases of the bladder? I should be glad if you will give me his address.—*E. A. F.*

Yes. See the Medical Directory for his address.

**Important Nursing Text-books.**

"The Nursing Profession: How and Where to Train." 2s. net; 2s. 4d. post free.

"The Nurses' Dictionary." By Honnor Morten. 2s. post free.

"Nursing: a Complete Text-book of Medical, Surgical, and Monthly Nursing." By Percy Lewis, M.D. 3s. 6d., post free.

"Elementary Physiology for Nurses." By C. F. Marshall, M.D. 2s. post free.

"A Complete Handbook of Midwifery for Nurses." By J. K. Watson, M.D. 6s. net; 6s. 4d. post free.

The above works are obtainable of all booksellers, or direct from The Scientific Press, Limited, 28 & 29 Southampton Street, Strand, London, W.C.

### For Reading to the Sick.

"THE ANGEL OF HIS PRESENCE SAVED THEM."

(*Isa.* lxiii. 9.)

O ye whose days are dark with grief,
Who ofttimes pray that life be brief,—
Look upward from the doubt and fear:
In all your sorrow shall appear
The angel of His presence here.

He hath redeemed—He will redeem;
His love to thee shall greater seem
For message of the pain, I ween:
"Within thy heart," the message tells,
"The angel of His presence dwells."

He was their Saviour—aye, in days
When men scarce understood His ways,
Yet dimly saw His glory rays:
He was their Saviour—in Love's name
The angel of His presence came.

And though thy days be long or brief,
Know that God's love o'ercrowns all grief
(His love means man's assured relief):
And when to thee there comes the end,
He will His angel's presence send.

*Whittier.*

If our eyes were opened, we should see ourselves, as did the servant of Elisha, surrounded with the armies of Heaven; we should see our daily life, with all its cares and troubles and anxieties, stretching away, like a great ladder, into heaven, and angels ascending and descending on it.

We should be reverent, pure, holy, and good, because we love God; because we fear His terrors and look for His glorious appearing, but also "because of the Angels." We should count it among the restraints, as it is undoubtedly among the consolations, of our spiritual life, that we are come "to an innumerable company of Angels." And while the world in its practical Sadduceeism says that there is no Resurrection, neither angel nor spirit, we shall, in this point at least, agree with the stricter religion of the ancient Pharisee, and in faith, life, and precept, to our intense comfort, confess both.—*Canon Newbolt.*

To the Christian the door of suffering opens the secret place of his Lord; he will enter there and learn of Him. It becomes the mainspring of all action, and exerts its influence over all the thoughts and feelings of the daily life; but it works silently, secretly; it holds communion with the invisible, and lays the hand of faith on all God's wide pasture-land even here below the sanctifying influence of sorrow brings its own comfort with it; quietly but surely it transforms the tastes, the desires, and the hopes, and as sorrow throws its shadow over the attractions of earth it makes the future bright with what can never fade. The tastes and thoughts of heaven take root here below, and are in harmony with the fadeless joys that shall be revealed.

*Mrs. C. G. Campbell.*

The real question for all of us is, What shall we hereafter desire to have felt about that which God has withheld? what shall we desire to have done with that which by His gift we have, be it much or little? what shall we desire ourselves to be when we know that the end of life is close upon us? Most assuredly that question is vital: in answering it let us never forget that man's life—that in him which will not perish at death (his soul)—consisteth not in the abundance of the things which he possesseth.—*H. P. Liddon.*

